# Engineering quantitative stomatal trait variation and local adaptation potential by cis‐regulatory editing

**DOI:** 10.1111/pbi.14464

**Published:** 2024-10-18

**Authors:** Nicholas G. Karavolias, Dhruv Patel‐Tupper, Ana Gallegos Cruz, Lillian Litvak, Samantha E. Lieberman, Michelle Tjahjadi, Krishna K. Niyogi, Myeong‐Je Cho, Brian J. Staskawicz

**Affiliations:** ^1^ Innovative Genomics Institute Berkeley California USA; ^2^ Department of Plant and Microbial Biology UC Berkeley Berkeley California USA; ^3^ Howard Hughes Medical Institute University of California Berkeley California USA; ^4^ Molecular Biophysics and Integrated Bioimaging Division Lawrence Berkeley National Laboratory Berkeley California USA

**Keywords:** gene editing, developmental genetics, environmental adaptation, rice

## Abstract

Cis‐regulatory element editing can generate quantitative trait variation that mitigates extreme phenotypes and harmful pleiotropy associated with coding sequence mutations. Here, we applied a multiplexed CRISPR/Cas9 approach, informed by bioinformatic datasets, to generate genotypic variation in the promoter of *OsSTOMAGEN*, a positive regulator of rice stomatal density. Engineered genotypic variation corresponded to broad and continuous variation in stomatal density, ranging from 70% to 120% of wild‐type stomatal density. This panel of stomatal variants was leveraged in physiological assays to establish discrete relationships between stomatal morphological variation and stomatal conductance, carbon assimilation and intrinsic water use efficiency in steady‐state and fluctuating light conditions. Additionally, promoter alleles were subjected to vegetative drought regimes to assay the effects of the edited alleles on developmental response to drought. Notably, the capacity for drought‐responsive stomatal density reprogramming in *stomagen* and two cis‐regulatory edited alleles was reduced. Collectively our data demonstrate that cis‐regulatory element editing can generate near‐isogenic trait variation that can be leveraged for establishing relationships between anatomy and physiology, providing a basis for optimizing traits across diverse environments.

## Introduction

In a global food system threatened by the effects of climate change, stomata have emerged as a central target of engineering due to their seminal role in water loss and photosynthesis (Leakey *et al*., 2019). Efforts to adapt germplasm to variable environments often include modifications to stomatal characteristics including density and kinetics (Caine *et al*., 2019; Faralli *et al*., 2019; Kumar *et al*., 2021; Mega *et al*., 2019; Papanatsiou *et al*., 2019; Wang *et al*., 2014; Zhang *et al*., 2021). Generating stomatal variants adapted to diverse environments remains a highly sought‐after goal.

The influence of stomatal density on other stomatal morphological traits, gas exchange, intrinsic water use efficiency (iWUE) and drought tolerance has been shown to be significant in a broad array of species (Caine *et al*., 2019, 2022; Karavolias *et al*., 2023; Kumar *et al*., 2021; Pitaloka *et al*., 2022; Roche, 2015; Zhao *et al*., 2022). In rice specifically, reductions in stomatal density have been associated with increases in iWUE, water conservation and drought adaptation (Caine *et al*., 2019, 2023; Esmaeilzadeh‐Moridani *et al*., 2022; Karavolias *et al*., 2023; Kumar *et al*., 2021). Despite promising abiotic stress tolerance phenotypes, rice with reduced stomatal densities sometimes exhibit concomitant reductions of carbon assimilation which may lower overall productivity (Caine *et al*., 2019, 2022; Karavolias *et al*., 2023; Mohammed *et al*., 2019; Roche, 2015). Studies seeking to relate stomatal density with physiology have also been constrained by limited morphological variation and genetically heterogenous backgrounds that complicate the ability to parse the effects of anatomical variation from the effects of other genetic determinants (Caine *et al*., 2019, 2023; Karavolias *et al*., 2023; Kumar *et al*., 2021; Mohammed *et al*., 2019; Zhao *et al*., 2022). To effectively leverage stomatal density alterations for climate change resilience, greater insights into the physiological outcomes of these modifications are needed.

Gene editing in plant species spurred on by the introduction of CRISPR/Cas tools has been used to generate variation in a breadth of climate‐resilience traits, including stomata (Karavolias *et al*., 2023; Yin *et al*., 2017). The vast majority of successes to date have been achieved through gene knockouts (Karavolias *et al*., 2021). In some cases, a knockout‐based approach can provide sufficient trait variation while minimizing deleterious pleiotropic effects (Karavolias *et al*., 2021, 2023; Liu *et al*., 2021). However, this approach is limited to a small number of genes whose null phenotypes are not detrimental to overall plant fitness.

Editing *cis*‐regulatory elements is another attractive gene‐editing approach to generate novel variation. Targeting mutations to regulatory regions can fine tune the expression of genes with fewer pleiotropic effects relative to coding sequence mutations and can expand the range of potential phenotypic outcomes (Hendelman *et al*., 2021; Liu *et al*., 2021; Rodríguez‐Leal *et al*., 2017). Generating broad trait diversity by gene editing is especially pertinent to rice crops whose production landscapes encompass a broad range of environmental conditions with radically different water availability (Khush, 1997; Tuong and Bouman, 2003). Cultivated varieties of rice display a range of stomatal densities and morphologies corresponding to the environment where they are produced (Esmaeilzadeh‐Moridani *et al*., 2022; Kumar *et al*., 2021; Ohsumi *et al*., 2015). Beyond the potential for crop improvement, *cis*‐regulatory element editing can also help provide insights into the complex process of transcriptional regulation.

To generate variation in stomatal traits, we designed guide RNAs targeted to the promoter of the gene encoding STOMAGEN in rice. *STOMAGEN*, is a positive regulator of stomatal development that is dynamically expressed in the mesophyll (Hunt *et al*., 2010; Hunt and Gray, 2009; Karavolias *et al*., 2023; Shimada *et al*., 2011; Sugano *et al*., 2010). The peptide encoded by *AtSTOMAGEN* binds the ERECTA (*ER*) family receptors and co‐receptor TOO MANY MOUTHS (*TMM*) in *Arabidopsis thaliana* facilitating downstream development (Hepworth *et al*., 2018; Lee *et al*., 2015; Shimada *et al*., 2011; Sugano *et al*., 2010). Knockouts of *OsSTOMAGEN* in rice yield an eight‐ and fivefold reduction in abaxial stomatal density in the IR64 and Nipponbare backgrounds, respectively (Karavolias *et al*., 2023; Yin *et al*., 2017). *OsSTOMAGEN* was selected as a target for cis‐regulatory element editing to fine‐tune stomatal density as an alternative to the severe reductions associated with knockouts.

The variation generated in this study was used to interrogate the structure–function relationships of stomatal density and physiology and the capacity of *cis*‐regulatory mutagenesis to fine‐tune stomatal density. Finally, we consider stomatal density reprogramming under vegetative drought stress to assess the impacts of *cis*‐regulatory element editing on environmental response. We apply these findings towards a framework to understand how approaches in promoter editing can be used to establish quantitative trait variation in elite germplasm for adaptation to broad and dynamic environments.

## Methods

### Plant growth conditions

Rice cultivar Kitaake (*O. sativ*a ssp. japonica) seeds were germinated and grown for 8 days in a petri dish with 20 mL of water in a Conviron growth chamber at 28 °C for day‐length periods of 16 h in 100 μmol photons m^−2^ s^−1^ of light and 80% relative humidity. Seedlings were transferred to a soil mixture comprised of equal parts turface (https://www.turface.com/products/infield‐conditioners/mvp) and sunshine mix #4 (http://www.sungro.com/professional‐products/fafard/).

Germinated seedlings used for stomatal phenotyping and growth chamber physiological assays were transferred to 10 cm, 0.75 L McConkey tech square pots and placed in growth chambers at 28 °C for day‐length periods of 16 h in 400 μmol photons m^−2^ s^−1^ of light and 80% relative humidity.

Plants designated for greenhouse measurements were placed in setpoints of 27 °C day/22 °C night at ambient light conditions in 15.2 cm, 1.8 L pots. All plants were fertilized with 125 mL of 1% w/v iron solution 1‐week post‐transplant. 1 L of 5% w/v JR Peter's Blue 20–20–20 fertilizer (https://www.jrpeters.com/) was added to each flat at 3‐ and 11‐weeks post‐germination. Data presented in the main figures are from greenhouse‐grown plants. Some experiments were repeated on plants grown in chambers.

### Identifying putative transcription factor binding sites, conserved non‐coding sequences, regions of open chromatin and H3K27ac DNA interaction regions

Conserved non‐coding sequences within the Poaceae family were identified using mVista (Mayor *et al*., 2000). The sequences of the 2 kb region upstream of the translation start site of the homologue of *OsSTOMAGEN* from *Brachypodium distachyon*, *Hordeum vulgare*, *Setaria italica* and *Zea mays* were retrieved from Phytozome. Sequence IDs for homologues are listed in Table S1. A duplication of *OsSTOMAGEN* in Poaceae required the use of the gene tree generated by Phytozome to identify orthologs of *STOMAGEN* within each species assayed. All *STOMAGEN* copies also had the highest percent identity compared to *OsSTOMAGEN* relative to other paralogs of *STOMAGEN* in species assayed. ATAC‐seq and ChIP‐seq data of *OsSTOMAGEN* were extracted from the publicly available RiceENCODE database (http://glab.hzau.edu.cn/RiceENCODE/pages/Browser.html) (Xie *et al*., 2021).

### Generation of edited lines

Forward and reverse strand guide sequences with relevant sticky ends amenable for Golden Gate cloning were ordered from Integrated DNA Technology (IDTdna.com). Guide sequences are listed in Table S3. Guides are arranged in order from most distal to proximal of the *OsSTOMAGEN* transcription start site. Equal volumes of 10 mm primers were annealed at room temperature. Golden Gate cloning was used to insert all eight guides simultaneously into the entry clone containing the tracrRNA and U3 promoter. LR clonase reactions were used to insert the entry clone into destination vectors for biolistic transformation and *Agrobacterium‐*mediated transformation. The guide for targeting the coding sequence of *OsSTOMAGEN* was selected to minimize off‐targeting, using CRISPR‐P 2.0 (http://crispr.hzau.edu.cn/CRISPR2/).

### Plant material and culture of explants

Mature seeds of rice (*O. sativa* L. japonica cv. Kitaake) were de‐hulled and surface‐sterilized for 20 min in 20% (v/v) commercial bleach (5.25% sodium hypochlorite) and 1% of Tween 20. Three washes in sterile water were used to remove residual bleach from seeds. De‐hulled seeds were placed on callus induction medium (CIM) medium [N6 salts and vitamins (Chu *et al*., 1975), 30 g/L maltose, 0.1 g/L myo‐inositol, 0.3 g/L casein enzymatic hydrolysate, 0.5 g/L L‐proline, 0.5 g/L L‐glutamine, 2.5 mg/L 2,4‐D, 0.2 mg/L BAP, 5 mm CuSO_4_, 3.5 g/L Phytagel, pH 5.8] and incubated in the dark at 28 °C to initiate callus induction. Six‐ to 8‐week‐old embryogenic calli were used as targets for transformation.

### *Agrobacterium*‐mediated transformation

Embryogenic calli were dried for 30 min prior to incubation with an *Agrobacterium tumefaciens* EHA105 suspension (OD_600_ = 0.1) carrying the binary vector for editing the *OsSTOMAGEN* promoter. After a 30 min incubation, the *Agrobacterium* suspension was removed. Calli were then placed on sterile filter paper, transferred to co‐cultivation medium [N6 salts and vitamins, 30 g/L maltose, 10 g/L glucose, 0.1 g/L myo‐inositol, 0.3 g/L casein enzymatic hydrolysate, 0.5 g/L L‐proline, 0.5 g/L L‐glutamine, 2 mg/L 2,4‐D, 0.5 mg/L thiamine, 100 mm acetosyringone, 3.5 g/L Phytagel, pH 5.2] and incubated in the dark at 21 °C for 3 days. After co‐cultivation, calli were transferred to resting medium [N6 salts and vitamins, 30 g/L maltose, 0.1 g/L myo‐inositol, 0.3 g/L casein enzymatic hydrolysate, 0.5 g/L L‐proline, 0.5 g/L L‐glutamine, 2 mg/L 2,4‐D, 0.5 mg/L thiamine, 100 mg/L timentin, 3.5 g/L Phytagel, pH 5.8] and incubated in the dark at 28 °C for 7 days. Calli were then transferred to the selection medium [CIM plus 250 mg/L cefotaxime and 50 mg/L hygromycin B] and allowed to proliferate in the dark at 28 °C for 14 days. Well‐proliferating tissues were transferred to CIM containing 75 mg/L hygromycin B. The remaining tissues were subcultured at 3–4 week intervals on a fresh selection medium. When a sufficient amount (about 1.5 cm in diameter) of the putatively transformed tissues was obtained, they were transferred to regeneration medium [MS salts and vitamins (Murashige and Skoog, 1962), 30 g/L sucrose, 30 g/L sorbitol, 0.5 mg/L NAA, 1 mg/L BAP, 150 mg/L cefotaxime] containing 40 mg/L hygromycin B and incubated at 26 °C, 16‐h light, 90 μmol photons m^−2^ s^−1^. When regenerated plantlets reached at least 1 cm in height, they were transferred to rooting medium [MS salts and vitamins, 20 g/L sucrose, 1 g/L myo‐inositol, 150 mg/L cefotaxime] containing 20 mg/L hygromycin B and incubated at 26 °C under conditions of 16‐h light (150 μmol photons m^−2^ s^−1^) and 8‐h dark until roots were established and leaves touched the Phytatray™ II lid (Sigma‐Aldrich, St. Louis, MO). Plantlets were then transferred to the soil.

### Validation of edits in promoter and coding sequence

T_0_ plants targeted for edits in Os*STOMAGEN* promoter were evaluated using PCR to amplify the entire 2 kb region upstream of the translation start site. To account for the possibility of heterozygous promoter mutations, PCR products were subcloned into Zero Blunt™ TOPO™ (Thermo Fisher, Waltham, MA). A total of 15 *E. coli* transformants were miniprepped and sequenced using the primers listed in Table S2. Seeds from T_0_ plants with heterozygous promoter mutations were genotyped using the same method in the T_1_ generation to isolate homozygous promoter mutations. Segregation of the T‐DNA cassette was verified and only seed from Cas9‐free, homozygousT_2_ plants were used for experimental data collection.

Coding sequence mutations in *OsSTOMAGEN* were evaluated using PCR to amplify the region of interest (Table S3). PCR products were Sanger sequenced. Sequence data were analysed using the Synthego ICE tool (https://ice.synthego.com/#/) to detect alleles present (Conant *et al*., 2022). Only lines with homozygous frame‐shift mutations were retained for downstream experiments.

### Phenotyping stomatal density and size

Stomatal densities were recorded from epidermal impressions of leaves using nail polish peels (Kusumi *et al*., 2017). Impressions were made on the widest section of 32‐day‐old fully expanded leaves of each biological replicate in well‐watered and drought conditions. Images were taken using a Leica DM5000 B epifluorescence microscope at 10× magnification. Three images were collected per stomatal impression and density per image was averaged. The number of stomata in a single stomatal band was counted and the area of each band was measured (Huang *et al*., 2019). Stomatal densities were calculated by dividing stomatal counts by stomal band area (mm^2^). Stomatal densities of fifth leaf abaxial tissues were assayed for each allele.

Epidermal peels for use in visualizing stomatal complex size of 40‐day‐old plants were produced using a razor blade on the adaxial leaf to remove tissues above the abaxial epidermal layer. Images of individual stomata at 100× magnification were captured. Guard cell length was measured using ImageJ. A total of 30 individual stomata collected from a total of five biological replicates of each genotype were measured.

### Quantifying *OsSTOMAGEN* transcript abundance

Total RNA was extracted from plants with the Qiagen Total RNAeasy Plant Kit at three developmental stages: 5 days after germination, from the basal 2.5 cm of the youngest developing leaf on 32‐day‐old plants and 2.5 cm from the leaf tip of the flag leaf of the primary tiller from 64‐day old plants for each promoter allele and wild type. For comparisons of transcript abundance in vegetative drought, total RNA was also extracted from the basal 2.5 cm of the youngest developing leaves of 32‐day‐old plants after a 5‐day vegetative drought stress was imposed. Flats of plants to be droughted were drained of all water 5 days prior to a sampling.

RNA was reverse transcribed using the QuantiTect™ reverse transcription kit to generate first‐strand cDNA. Quantitative reverse transcription PCR was performed using FAST SYBR on Applied Biosystem's QuantStudio 3 thermocycler. Relative expression levels were calculated by normalizing to the average of rice housekeeping genes, *UBQ5* (LOC_Os01g22490) and *eEF‐1A* (LOC_Os03g08020) (Jain *et al*., 2018). Primers used for qPCR are listed in Table S1. Relative log‐fold expression was calculated using the 2^−ΔΔCT^ method using the ‘Do my qpcr’ web tool (Tournayre *et al*., 2019). For flag leaves, seedlings and developing leaves, all comparisons were made to the wild type. For comparisons of well‐watered to vegetative drought‐developing leaves, all expression is relative to wild‐type well‐watered. For genotype‐specific comparisons across tissue types, comparisons were made to flag leaves.

### Photosynthesis and stomatal conductance assays

Physiological assays in Figures 2 and 3 were conducted on the fully expanded leaf five of 32‐day‐old plants. Data for Figures 2 and 3 were captured using an infrared gas analyser (LI6800XT, LI‐COR, Lincoln, NE) with chamber conditions set to: light intensity 1500 μmol photons m^−2^ s^−1^ (90% red light, up to 10% blue light [max. 40 μmol photons m^−2^ s^−1^]); leaf temperature 25 °C; flow rate 500 μmol s^−1^; relative humidity 50% and CO_2_ concentration of sample 400 ppm. Each biological replicate was allowed to acclimate for 15 min prior to data collection. Leaves from which gas exchange data was captured were marked and subsequently phenotyped for stomatal density.

Measurements of gas exchange on vegetatively droughted plants were conducted using the methods described above. Drought was applied by draining the flats of plants entirely. Gas exchange measurements were taken on the fifth day of the drought. The length of the drought was established by noting the number of days to onset of leaf rolling in pilot studies.

### Fluctuating light assay

Plants were dark acclimated for 1 h prior to the light induction assay. Plants were then subjected to the following conditions leaf temperature 25 °C; flow rate 500 μmol s^−1^; relative humidity 50% and CO_2_ concentration of sample 400 ppm. The light intensity was initially set to 0 μmol photons m^−2^ s^−1^ for 3 min during which F_v_/F_m_ recordings were made. Only plants with F_v_/F_m_ greater than 0.77 were used in this assay. After 3 min, the light intensity was increased to 100 μmol photons m^−2^ s^−1^ for 15 min. Plants were then kept at 1500 μmol photons m^−2^ s^−1^ (90% red light, up to 10% blue light [max. 40 μmol photons m^−2^ s^−1^]) for 78 min, followed by 100 μmol photons m^−2^ s^−1^ for 15 more minutes. Gas exchange data were logged every 10 s.

A curve fitting each data point to its adjacent point was produced and the area under this curve was calculated for stomatal conductance and for carbon assimilation. The integrated carbon assimilation value was divided by the integrated stomatal conductance value to generate a measurement of intrinsic water use efficiency in this dynamic range. A one‐phase decay model was fitted to the stomatal conductance data restricted to the region of the curve immediately after the light‐to‐dark transition. The rate constant of this curve was calculated and used to estimate stomatal closure rates.

### Stomatal density reprogramming in drought assay

The stomatal density modifications induced by the drought regime used identical phenotyping methods as described above to measure densities. In this assay, the vegetative drought was applied by removing all water from the flats for 7 days when plants were at the four‐leaf developmental stage. On the final day of the drought, the youngest emerging leaf among the three largest tillers was marked using a plastic tie at the leaf base. To ensure that the leaf measured was one that developed during drought, only the youngest leaves that emerged during the course of the 7‐day drought regime were considered. Droughted flats were fully rehydrated at day seven and plants were allowed to continue developing. A well‐watered regime was applied to plants grown alongside droughted plants. Epidermal impressions were made on fully expanded fifth leaves from well‐watered and droughted plants and measured according to previous methods (Huang *et al*., 2019).

### Graphs and statistics

Plots were generated using Adobe Illustrator 24.1. and GraphPad Prism 9.5. Statistics were computed in R studio. Comparisons of multiple groups were conducted using ANOVA and Tukey's honest significant difference (HSD) post‐hoc tests.

## Results

### Bioinformatic analyses inform promoter regions for editing

Signatures of putative significant regulatory motifs in the promoter of *OsSTOMAGEN* were identified to inform guide design. Overlaying conserved non‐coding sequences (CNS), ATAC‐seq and ChIP‐seq data identified possible genomic signatures to target by cis‐regulatory mutagenesis (Figure S1) (Rodríguez‐Leal *et al*., 2017; Zhou *et al*., 2023). The greatest conservation among non‐coding sequences was observed in *Brachypodium distachyon* relative to other family members.

**Figure 1:**
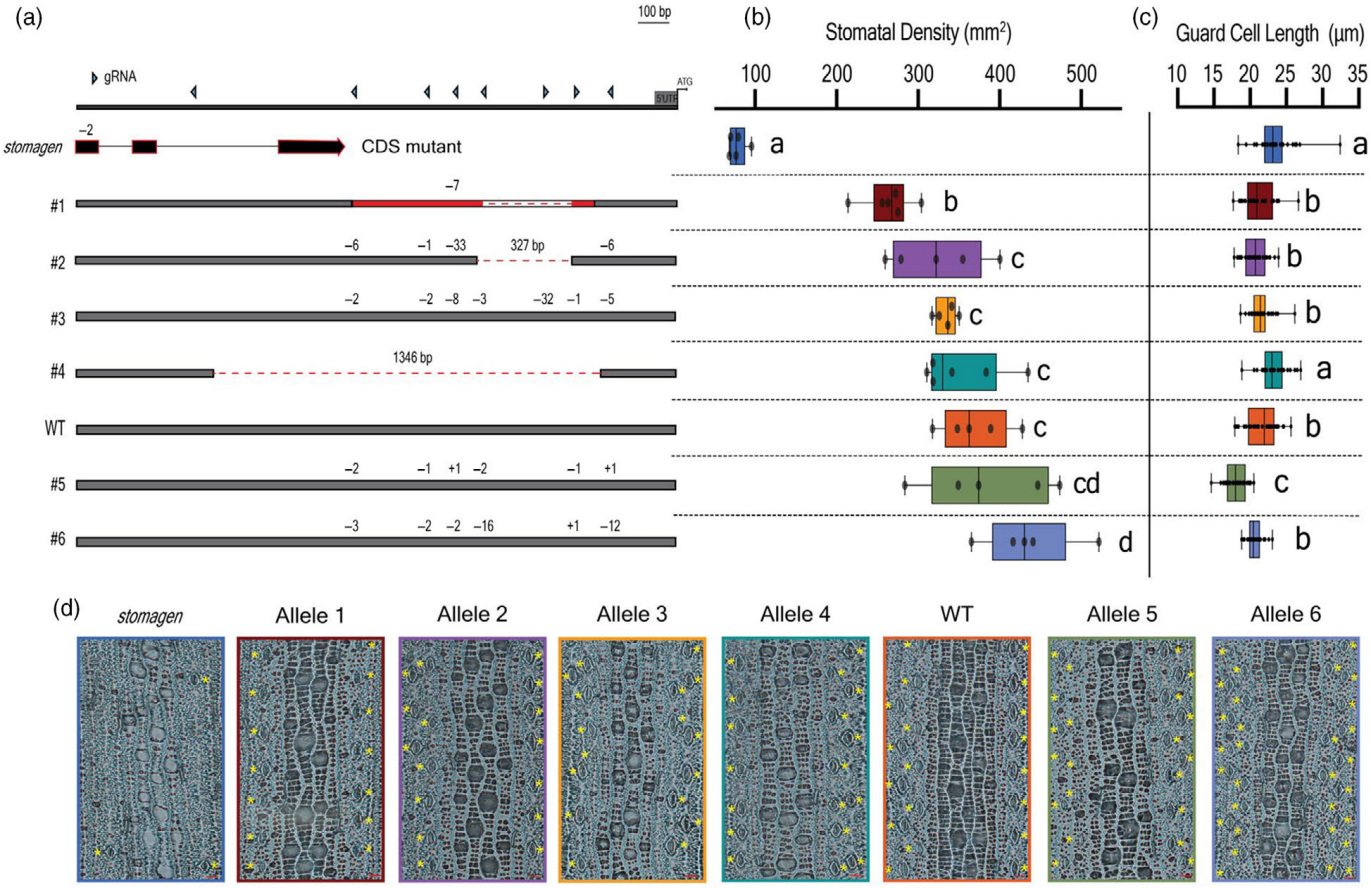
Stomatal density and variation in size across gene‐edited *OsSTOMAGEN* promoter alleles. (a) The genotype of each promoter allele and the *stomagen* CDS mutant. Red‐shaded regions indicate inversion. The size of each indel is listed above the cut site. (b) Box‐and‐whisker plot of the stomatal density of each allele assayed. (c) Box‐and‐whisker plots of guard cell length of each allele assayed. (d) Representative images of epidermal cells of each genotype assayed. Yellow asterisks are placed adjacent to each stoma. Red scale bars represent 20 μm. In the box‐and‐whisker plots, the centre horizontal indicates the median, upper and lower edges of the box are the upper and lower quartiles and whiskers extend to the maximum and minimum values within 1.5 interquartile ranges. Each dot represents a biological replicate. Letters indicate a significant difference between means (*P* < 0.05, one‐way ANOVA Tukey HSD post‐hoc test). Barplots mean is represented with error bars showing the standard error of the mean. Asterisks represent a significant difference in expression relative to wild type (*P* < 0.05, one‐way ANOVA Tukey HSD post‐hoc test).

The eight guide RNAs selected for targeting the upstream region of *OsSTOMAGEN* are indicated by blue triangles in Figure S1. All selected guides correspond to at least one signature detected in the bioinformatic analysis, prioritizing regions where ATAC‐seq, ChIP‐seq and CNS peaks coincided. Alleles generated (Figure 1) represent editing outcomes consistent with both simultaneous and asynchronous activity of multiple guides. A single guide construct was used to generate coding sequence mutations in the first exon *OsSTOMAGEN* (Figure S2).

### *OsSTOMAGEN* promoter alleles alter stomatal density and morphology

The phenotypic implications of *OsSTOMAGEN* promoter and coding sequence mutations were first assessed by measuring stomatal density. The stomatal density of the *stomagen* knockout in *Oryza sativa* cv. Kitaake exhibited an 80% reduction of wild‐type stomatal density, consistent with knockout phenotypes observed in cv. Nipponbare and cv. IR64 (Karavolias *et al*., 2023; Yin *et al*., 2017) (Figure 1b; Figure S3). The panel of promoter‐edited alleles exhibited a broad diversity of stomatal densities including four lines with densities lower than—and two lines with densities greater than—wild type (Figure 1; Figure S3). The ranked order of stomatal density alleles remained the same in greenhouse and growth chamber conditions (Figure 1; Figure S3).

Beyond density, guard cell length was measured as a proxy of stomatal size. Variation in stomatal size was observed in the panel of *OsSTOMAGEN* alleles assayed (Figure 1). Deviations from a linear relationship between stomatal density and size suggest that stochasticity in guard cell length could be related to promoter mutations (Figure S4). Thus, broad quantitative variation in stomatal density and size was successfully established by coding sequence and promoter editing of *OsSTOMAGEN*.

### *OsSTOMAGEN* transcript abundance across multiple tissue types does not correlate with stomatal density phenotypes

The transcript abundance of *OsSTOMAGEN* across multiple tissue types was subsequently assessed in promoter alleles to determine the relationships between expression and phenotype. *OsSTOMAGEN* transcript abundance was measured in each promoter allele relative to wild‐type expression in flag leaves, seedlings and developing leaves (Figure S5). In developing leaves where *OsSTOMAGEN* exhibits the greatest expression in wild type, there is no obvious co‐linearity of stomatal density and expression level. Similarly, in flag leaves and seedlings, there is no obvious relationship between density and expression levels (Figure S5). The ratio of STOMAGEN expression levels across tissue types varied within individual promoter alleles as well (Figure S6).

### Near‐isogenic panel reveals relationships between stomatal density and gas exchange physiology

**Figure 2:**
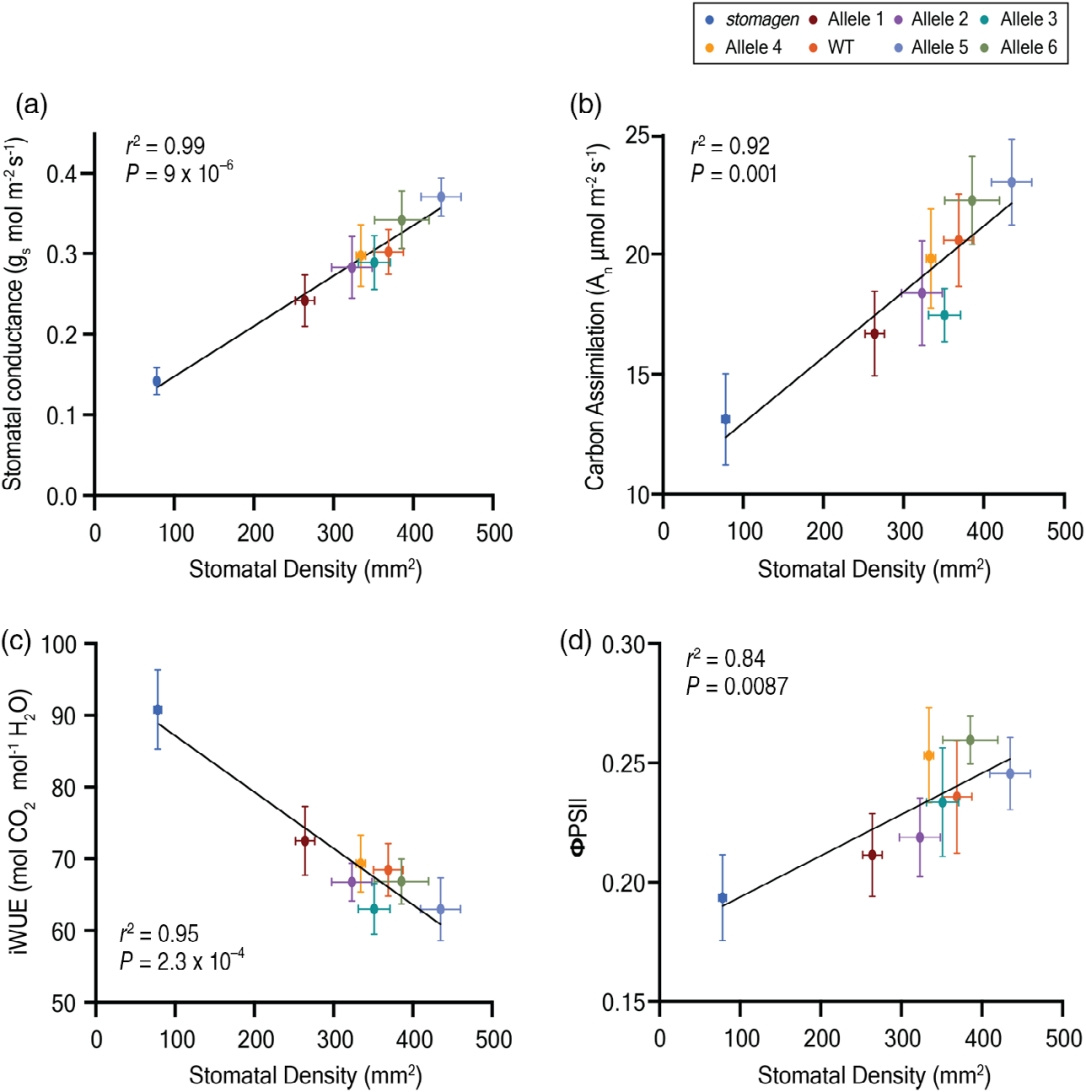
Stomatal morphological variation corresponds to gas exchange variation in a near‐isogenic panel. Linear regression of stomatal density and (a) stomatal conductance, (b) carbon assimilation, (c) intrinsic water‐use efficiency and (d) ΦPSII. The correlation coefficient (*r* ^2^) and *P*‐value of each correlation are noted in each panel. The mean and standard error of the mean of eight biological replicates are reported.

The anatomical diversity of stomatal density warranted further assessments of physiology within this near‐isogenic context. The relationships of stomatal density to carbon assimilation, stomatal conductance, iWUE and the quantum yield of photosystem II (ΦPSII) were established leveraging the diversity inherent to the panel (Figure 2). Carbon assimilation refers to the rate of carbon dioxide uptake/capture by the leaf, stomatal conductance is a measurement of the rate of water vapour flow via stomata, iWUE is the ratio of carbon assimilation to stomatal conductance and ΦPSII represents the operating efficiency of photosystem II (Faralli *et al*., 2019; Yin *et al*., 2010). A strong, positive, linear association between stomatal density and steady‐state carbon assimilation, stomatal conductance and ΦPSII at a light intensity of 1500 μmol photons m^−2^ s^−1^ was observed (Figure 2). Likewise, a strong, negative, linear association between stomatal density and iWUE was established (Figure 2).

### Fluctuating light assay reveals dynamic gas exchange phenotypes across the stomatal density panel

**Figure 3:**
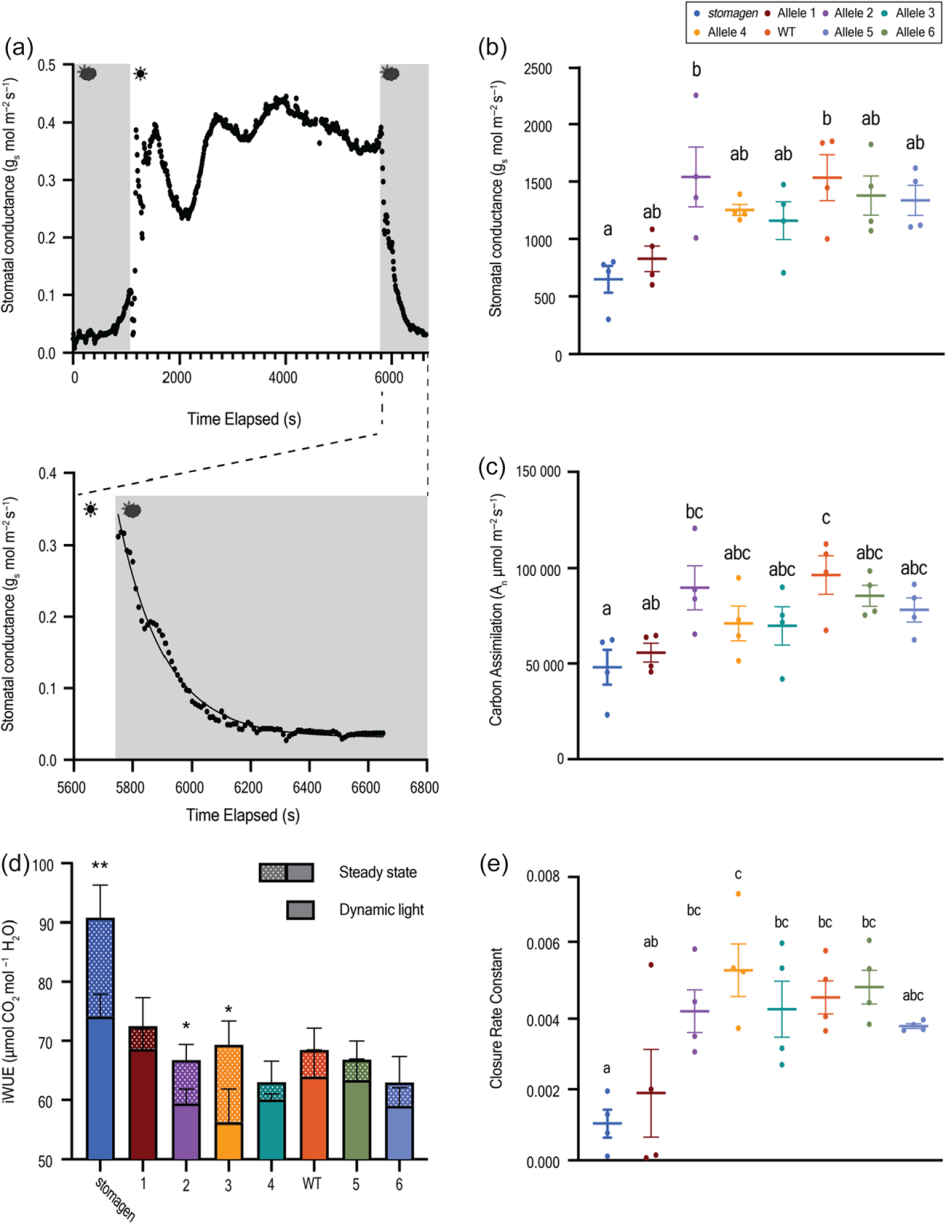
Physiological response to dynamic environmental conditions. (a) Representative WT stomatal conductance response curve to fluctuating light with grey blocks representing low light and white blocks representing high light. The lower inset is a representative one‐phase decay curve fit to the second low‐light region of the curve. Dotplot of (b) cumulative stomatal conductance and (c) cumulative carbon assimilation across the entire regime, as calculated by area under the curve. (d) A stacked barplot of iWUE was calculated from steady‐state and fluctuating light conditions. Solid blocks refer to fluctuating light conditions with patterned blocks superimposed to reflect steady‐state values. (e) Closure rate constant among alleles assayed in fluctuating light regimes, derived from the one‐phase decay curve fit to the stomatal conductance data following return from high light to low light. In the dotplots, each dot represents a biological replicate with bars indicating the mean and standard error of the mean. Letters indicate a significant difference between means (*P* < 0.05, one‐way ANOVA Tukey HSD post‐hoc test). In the barplot, the mean is represented with error bars showing the standard error of the mean. * and ** represent *P* values of ≤0.1 and ≤0.05, respectively. Significance values were calculated from the *t*‐test with Welch's correction.

To further investigate the relationship of stomatal variants to physiology, gas‐exchange phenotypes were monitored at high temporal resolution in response to fluctuating light (Figure 3a; Figure S8). We observed a multimodal response in stomatal conductance in response to high light, consistent with previous reports (Xiong *et al*., 2018). Similar to the steady‐state phenotypes reported in Figure 2, stomatal conductance and carbon assimilation maintain a positive correlation with stomatal density and a negative correlation with iWUE (Figure 3). However, the cumulative iWUE, calculated from the area under the curve in dynamic light conditions, was lower than the steady‐state measurement in all genotypes. Additionally, the difference among genotypes in the dynamic light regime was less pronounced (Figure 3). To further resolve this difference in steady‐state and dynamic light phenotypes, the rate of stomatal conductance reduction in response to transition from high light to low light was quantified (Figure 3). A one‐phase decay model was fit to the stomatal conductance data immediately following the low light transition for each biological replicate and rate constants were derived as a proxy for the stomatal closure rate. Low density, large stomata *stomagen* knockout exhibited the slowest closure rate constant, with variation in closure rate constants generally varying with a stomatal density among other genotypes (Figure 3).

### Capacity for physiological and developmental response to abiotic stress differs among promoter alleles

**Figure 4:**
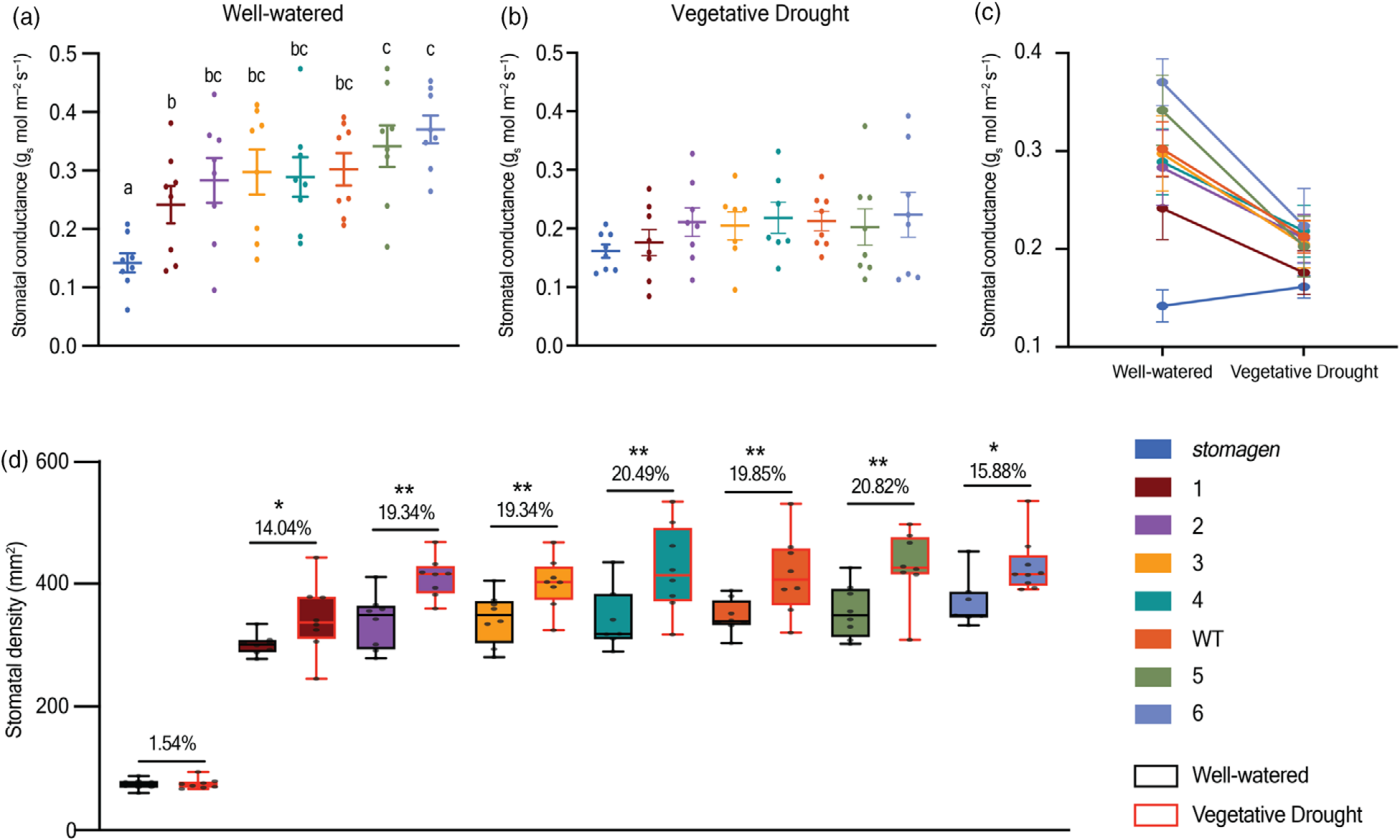
Physiological and developmental response of stomatal variants to vegetative drought. Dotplot of stomatal conductance in (a) well‐watered plants and (b) after a 5‐day vegetative drought. (c) A reaction normalization plot of stomatal conductance in each watering regime. (d) Changes in stomatal density in response to vegetative drought. The percent increase of vegetative drought stomatal density relative to well‐watered is reported above each genotype. In the dotplots, each dot represents a biological replicate with bars indicating the mean and standard error of the mean. Letters indicate a significant difference between means (*P* < 0.05, one‐way ANOVA Tukey HSD post‐hoc test). In the box‐and‐whisker plot, the centre horizontal indicates the median, upper and lower edges of the box are the upper and lower quartiles and whiskers extend to the maximum and minimum values within 1.5 interquartile ranges. The barplot shows means and error bars represent the standard error of the mean. In (d) black and red outlines represent well‐watered and vegetative drought, respectively. * represents a *P* value <0.1, <0.05 and ** represents a *P* value <0.05 (Student's t‐test).

The response of stomatal conductance in stomatal morphological variants under vegetative drought was also assayed. The variation in stomatal conductance identified in well‐watered conditions was nearly eliminated after imposing a 5‐day vegetative drought (Figure 4). All alleles besides *stomagen* exhibited an overall reduction in stomatal conductance in response to vegetative drought, whereas *stomagen* maintained a consistently low overall stomatal conductance regardless of drought status (Figure 4).

A similar drought assay was imposed on a separate cohort of plants to monitor drought‐responsive changes in stomatal density. A moderate vegetative drought resulted in stomatal density increases in the wild‐type background (Figure 4). This finding is consistent with previous studies where a vegetative drought was applied to a grass species (Meng *et al*., 1999; Xu and Zhou, 2008). The *stomagen* knockout, however, exhibited very limited stomatal density reprogramming response after drought (Figure 4). Most *OsSTOMAGEN* promoter alleles exhibited a wild‐type increase in stomatal density after drought stress (Figure 4). However, in alleles 1 and 6, the capacity for drought‐responsive stomatal density reprogramming may be diminished (Figure 4).

*OsSTOMAGEN* transcript abundance after drought stress treatment was analysed to discern the effect of promoter edits on expression. A drought‐responsive transcriptional increase of *OsSTOMAGEN* in wild‐type developing leaves was consistent with the observed increase in stomatal density (Figure S9). Likewise, promoter allele 1 also exhibited an increase in *OsSTOMAGEN* transcript abundance, despite exhibiting attenuated stomatal density reprogramming. No *OsSTOMAGEN* expression increases after vegetative drought were detected in any other promoter allele and a reduction in expression was observed in Allele 3. Consistent with previous *OsSTOMAGEN* expression data, we observed limited co‐linearity between expression levels and phenotype in response to vegetative drought, despite a consistent stomatal density increase among most promoter alleles (Figure S5).

## Discussion

Editing cis‐regulatory elements provides the opportunity to rapidly generate novel quantitative variation within a trait of interest. Application of this approach to *OsSTOMAGEN*, a positive regulator of stomatal density, produced an array of promoter alleles representing 70%–120% of wild‐type stomatal density. A multiplexed editing approach leveraging publicly available bioinformatic datasets to select gRNAs was pursued. Current methods can support the selection of guides that may generate quantitative variation, but cannot necessarily predict the specific directionality or magnitude of edits on transcriptional or phenotypic outcomes (Zhou *et al*., 2023). Nonetheless, the incorporation of these bioinformatic signatures enabled the selection of guides that ultimately had a substantial impact on phenotypic outcomes.

Notably, a higher stomatal density was observed in two edited promoter alleles. Hypermorphic phenotypes generated by promoter editing to date have been associated with genomic rearrangements (Lu *et al*., 2021; Patel‐Tupper *et al*., 2024). Here we report two hypermorphic alleles produced by indels alone, suggesting that the capacity to generate gain‐of‐function variants may be possible without larger perturbations and may be highly target‐gene dependent.

One of the unique attributes of these two alleles is a fully unedited region that corresponds to a CNS peak shared with the C3 grasses *B. distachyon* and *H. vulgare*. The transition between C3 and C4 photosynthesis includes a marked reduction of stomatal density linked to reductions in *STOMAGEN* expression (Zhao *et al*., 2022). It is thus possible that this C3‐specific CNS peak contributes positively to the stomatal density phenotype and future work could interrogate the mechanistic basis of how this putative cis‐regulatory element contributes to stomatal phenotypes. This insight also suggests cis‐regulatory editing programmes may benefit from considerations of evolutionary divergence when selecting target regions to facilitate an improved understanding of how *cis*‐regulatory elements contribute to trait emergence.

To understand the effects of edited promoter alleles on gene expression, the transcript abundance of *OsSTOMAGEN* was measured across various developmental stages and abiotic stresses. There were some differences in *OsSTOMAGEN* expression levels among promoter alleles; however, these differences in mRNA levels did not correlate to stomatal density outcomes. This finding is consistent with some previous applications of promoter editing where expression levels of targeted genes were not correlative with phenotypic outcomes (Rodríguez‐Leal *et al*., 2017; Song *et al*., 2022; Zhou *et al*., 2023). In examples where expression levels do not predict phenotypes, mis‐regulation of expression of targeted genes may occur in highly discrete spatial or temporal zones that can be difficult to observe. In these cases, gene expression may be insufficient as a proxy for phenotype, highlighting the importance of robust phenotyping of gene‐edited phenotypes.

Our stomatal density panel also enhanced our ability to resolve density versus gas exchange phenotypes. A strong, positive, linear, relationship between stomatal density and stomatal conductance and carbon assimilation was established, consistent with previous reports. Likewise, a strong, negative linear association of stomatal density and iWUE was found (Caine *et al*., 2019; Karavolias *et al*., 2023; Ohsumi *et al*., 2015; Pitaloka *et al*., 2022). Interestingly stomatal density was found to have a positive, linear relationship with ΦPSII. Transpiration via stomata plays an essential role in evaporative cooling under peak diurnal temperatures and irradiances. Long‐term or repeated exposure to heat stress, exacerbated by low stomatal densities, may contribute to this relationship between density and ΦPSII (Yin *et al*., 2010). Our CRISPR near‐isogenic lines provide a major advantage in overcoming previous limitations of studies relying on natural variation in stomatal density phenotype across diverse germplasm, resolving discrete structure–function relationships.

A fluctuating light assay revealed additional structure–function relationships beyond traditional steady‐state measurements. Steady‐state measurements may overstate the gains in water‐use efficiency of reduced stomatal density lines, as shown by the decrease in iWUE differences under dynamic, fluctuating light. Discrepancies may result from slower rates of stomatal closure in low density, larger stomata lines. These findings may reconcile previous data that show similar levels of water loss from lines with moderate and strong reductions in stomatal density (Caine *et al*., 2019; Karavolias *et al*., 2023). Engineering stomatal density reductions with simultaneous adjustments to stomatal size and kinetics may thus be an even more promising mechanism to generate WUE improvements in dynamic field conditions (Papanatsiou *et al*., 2019; Wang *et al*., 2014).

This panel also enhanced our ability to interrogate developmental plasticity under drought stress, which is known to cause stomatal density reprogramming (Hetherington and Woodward, 2003; Meng *et al*., 1999; Xu and Zhou, 2008). Consistent with previous reports of stomatal density shifts in drought stress, we find moderate increases in wild‐type stomatal density after the drought regime (Meng *et al*., 1999; Xu and Zhou, 2008). Stomatal density increases correlated with increases in *OsSTOMAGEN* expression in the wild type, contrary to trends observed in all but one gene‐edited allele. Stomatal density reprogramming capacity during abiotic stress may also be lower in the lowest (Allele 1) and highest (Allele 6) stomatal density alleles and is completely abolished in the *stomagen* mutant background despite the presence of the functional paralog of *OsSTOMAGEN*, EPFL10, in all gene‐edited lines. Thus, it is likely that the regulation of *OsSTOMAGEN* activity is a critical bottleneck during the drought acclimation response. There are however no apparently unique edits common to Alleles 1 and 6. *OsSTOMAGEN* mis‐regulation and resulting phenotypes observed among CRE edited lines indicate complex interactions of environmental stimuli with promoters which may be worthy of additional investigation.

Aside from differences in development, we also describe greater magnitude changes in drought‐responsive stomatal conductance as indicated by the reaction normalization plot (Figure 4c). Varied physiological and developmental responses to vegetative drought among promoter alleles may offer greater insights to allele compatibility in environments with varied water availabilities. Notably, rice production spans highly variable environmental conditions, especially with regards to water availability (Esmaeilzadeh‐Moridani *et al*., 2022; Kumar *et al*., 2021; Ohsumi *et al*., 2015). Varieties bred for upland and other high water‐scarcity environments exhibit moderately reduced stomatal densities relative to stomatal densities of higher‐water availability cultivars (Esmaeilzadeh‐Moridani *et al*., 2022; Kumar *et al*., 2021); whereas, high‐yielding cultivars bred in water‐replete environments exhibit greater stomatal densities relative to cultivars adapted for broader environments (Esmaeilzadeh‐Moridani *et al*., 2022; Kumar *et al*., 2021; Ohsumi *et al*., 2015). This extant variation suggests there may be a role for gene‐editing tools to accelerate local adaptation of elite varieties to diverse landscapes.

**Figure 5:**
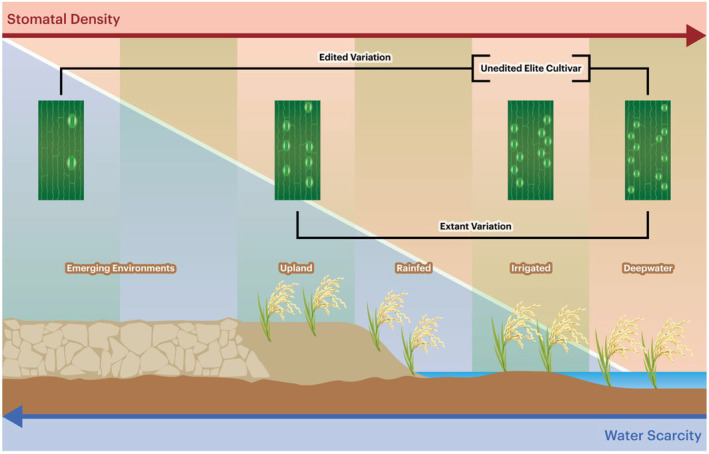
Stomatal morphological variation corresponds to current and emerging rice production schemes. Rice is produced in diverse environments with varying water availability. Extant stomatal morphological variants are adapted to specific rice production landscapes. The breadth of stomatal density variation achieved by gene editing exceeds natural variation and may provide the adaptive potential to improve productivity in emerging environments with high and variable levels of water scarcity.

Editing *OsSTOMAGEN* cis‐regulatory elements enabled the generation of alleles that covered and exceeded the range of stomatal density present across existing germplasm. Elite cultivars that are currently best adapted to narrow geographies due to limitations in a discrete set of traits could theoretically be optimized by cis‐regulatory element editing for compatibility to a much broader set of environments. Additionally, these variants may provide important fitness benefits in emerging environments increasingly constrained by resource availability, which may be increasingly common due to the effects of climate change (Figure 5). Taken together, our data offer an improved understanding of the opportunities of cis‐regulatory element editing in generating quantitative trait variation for broad and dynamic environments and an expansion of promoter editing as a tool to support local adaptation.

## Author contributions

NGK developed the project concept and coordinated research efforts. MT transformed and regenerated rice plants in the lab of MJC with technical support from MJC. Rice stomatal density phenotyping was led by NGK with support from AGC, LL and SAL. qPCR experiments were completed by NGK, AGC, LL and SAL. Physiological experiments were completed by NGK with technical advice from DPT. LICOR equipment was made available by KKN. All work was completed in the lab of BJS. The manuscript was drafted by NGK and edited by DPT.

## Supporting information


**Figure S1** Rational guide design approach for targeting the promoter of *OsSTOMAGEN*.
**Figure S2** Genotype of edited *stomagen* allele.
**Figure S3** Stomatal density variation of promoter alleles grown in the growth chamber.
**Figure S4** Linear regression of stomatal density and guard cell length.
**Figure S5** Tissue‐specific expression of *OsSTOMAGEN* among promoter alleles.
**Figure S6** Relative expression of *OsSTOMAGEN* in varying tissues within each promoter allele.
**Figure S7** Well‐watered greenhouse gas exchange measurements.
**Figure S8** Stomatal conductance response curves in fluctuating light.
**Figure S9** Drought responsive expression of *OsSTOMAGEN*.
**Table S1** Primer sequences
**Table S2** Guide sequences arranged from distal through proximal to translation start site
**Table S3** Summary of linear regressions

## Data Availability

The data that supports the findings of this study are available in the supplementary material of this article.
